# Redox environment is an intracellular factor to operate distinct pathways for aggregation of Cu,Zn-superoxide dismutase in amyotrophic lateral sclerosis

**DOI:** 10.3389/fncel.2013.00240

**Published:** 2013-11-27

**Authors:** Yoshiaki Furukawa

**Affiliations:** Laboratory for Mechanistic Chemistry of Biomolecules, Department of Chemistry, Keio UniversityYokohama, Japan

**Keywords:** SOD1, ALS, aggregation, disulfide bond

## Abstract

Dominant mutations in Cu,Zn-superoxide dismutase (SOD1) cause a familial form of amyotrophic lateral sclerosis (fALS). Misfolding and aggregation of mutant SOD1 proteins are a pathological hallmark of SOD1-related fALS cases; however, the molecular mechanism of SOD1 aggregation remains controversial. Here, I have used *E. coli* as a model organism and shown multiple distinct pathways of SOD1 aggregation that are dependent upon its thiol-disulfide status. Overexpression of fALS-mutant SOD1s in the cytoplasm of *E. coli* BL21 and SHuffle^TM^, where redox environment is reducing and oxidizing, respectively, resulted in the formation of insoluble aggregates with notable differences; a disulfide bond of SOD1 was completely reduced in BL21 or abnormally formed between SOD1 molecules in SHuffle^TM^. Depending upon intracellular redox environment, therefore, mutant SOD1 is considered to misfold/aggregate through distinct pathways, which would be relevant in description of the pathological heterogeneity of SOD1-related fALS cases.

## INTRODUCTION

Thiol-disulfide status is critical for functioning of many proteins (Sevier and Kaiser, 2002), and Cu,Zn-superoxide dismutase (SOD1) is one of such proteins in which formation of an intramolecular disulfide bond is required for folding into its enzymatically active conformation (Furukawa et al., 2004). An enzymatic function of SOD1 is to catalyze the removal of a toxic reactive oxygen species, superoxide anion (McCord and Fridovich, 1969), and activation steps of SOD1 *in vivo* include binding of a catalytic copper ion and a structural zinc ion and also formation of an intramolecular disulfide bond. Given that SOD1 isolated from *Bacillus subtilis* (Banci et al., 2005) and *Mycobacterium tuberculosis* (Spagnolo et al., 2004) lacks a copper and zinc binding site, respectively, metal binding seems to be dispensable for SOD1. In contrast, an intramolecular disulfide bond is conserved among all SOD1 proteins identified so far, implying its essential roles in physiological functions of SOD1.

Indeed, abnormalities in a thiol-disulfide status of SOD1 have been proposed as a pathological change in a familial form of amyotrophic lateral sclerosis (fALS) that is caused by dominant mutations in SOD1 (Rosen et al., 1993). For example, in transgenic mice expressing human SOD1 with a fALS mutation (G85R), two Cys residues (Cys57 and Cys146) of SOD1, which normally form an intramolecular disulfide bond, remained reduced (Jonsson et al., 2006). Aggregation of mutant SOD1 is a major pathological change in SOD1-related fALS cases (Bruijn et al., 1998), and inclusions reproduced in diseased mice have been shown to contain disulfide-reduced SOD1 proteins (Jonsson et al., 2006; Karch et al., 2009). *In vitro* studies have also shown increased susceptibility of a disulfide bond in several fALS-mutant SOD1 proteins toward a reducing agent (Tiwari and Hayward, 2003). Furthermore, reduction of a disulfide bond significantly decreased the thermostability and thus facilitated misfolding and aggregation of SOD1 *in vitro*, supporting important roles of a conserved disulfide bond in maintaining an aggregation-resistant structure of SOD1 (Furukawa and O’Halloran, 2005; Furukawa et al., 2008).

In contrast, increased oxidative stress has been reported in fALS patients (Barber and Shaw, 2010), and SOD1 appears to be one of intracellular targets susceptible to oxidative modifications (Guareschi et al., 2012). In transgenic mice expressing human SOD1 with fALS mutations, mutant SOD1 has been shown to form insoluble oligomers cross-linked *via* intermolecular disulfide bonds (Furukawa et al., 2006). *In vitro* experiments have also revealed that aggregation of mutant SOD1 is triggered by abnormal oxidation of histidine and tryptophan residues (Rakhit et al., 2002; Zhang et al., 2003). Under such oxidative conditions, a disulfide-reduced form of SOD1 would not stably exist. Furthermore, structural destabilization of SOD1 by pathogenic mutations has been recently reported to facilitate isomerization of a conserved intramolecular disulfide bond (Cys57–Cys146) into an intermolecular disulfide crosslink (Toichi et al., 2013). Taken together, reduction of a disulfide bond may not be a prerequisite for aggregation of SOD1 *in vivo*; rather, a redox environment surrounding SOD1 would determine how mutant SOD1 is misfolded and aggregated. Indeed, most (>70%) of intracellular SOD1 exist in the reducing environment of cytoplasm (Chang et al., 1988), but a small fraction (~3%) of SOD1 is also detected in the intermembrane space (IMS) of mitochondria (Okado-Matsumoto and Fridovich, 2001), which is considerably more oxidizing than cytoplasm (Hu et al., 2008). Intracellular SOD1 is thus considered to experience a broad range of redox environment, which would affect its folding/misfolding processes.

In this study, effects of intracellular redox environment on SOD1 aggregation have been examined in *Escherichia coli* as a model organism. Overexpression of heterologous proteins in bacteria such as *E. coli* often leads to the formation of insoluble aggregates called inclusion bodies, and inclusion bodies of several proteins have been shown to possess amyloid-like properties (Carrio et al., 2005; de Groot et al., 2009; Villar-Pique and Ventura, 2012). Cytoplasm of *E. coli* has been well known as strongly reducing environment (Hwang et al., 1992), while genetically modified *E. coli*, SHuffle^TM^, provides considerably oxidizing cytoplasm (Lobstein et al., 2012). FALS-mutant human SOD1 proteins in *E. coli* BL21 were found to exist as a disulfide-reduced state and form insoluble fibrillar aggregates. In contrast, expression of mutant SOD1 in the oxidizing cytoplasm of SHuffle^TM^ resulted in the formation of insoluble oligomers crosslinked *via* intermolecular disulfide bonds albeit with fibrillar morphologies. Depending upon the intracellular redox environment, therefore, mutant SOD1 proteins form insoluble aggregates with distinct properties, suggesting roles of organelle-specific misfolding pathways of mutant SOD1 in fALS pathomechanism.

## RESULTS AND DISCUSSION

### SOD1 AGGREGATES UNDER REDUCING ENVIRONMENT OF *E. coli* CYTOPLASM

**FIGURE 1:**
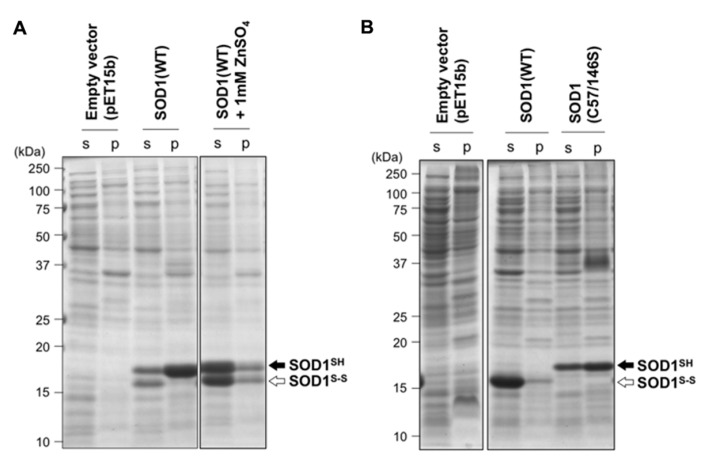
**Redox environment of *E. coli* cytoplasm influences the aggregation propensities of SOD1 by modulating its thiol-disulfide status. (A)** *E. coli* BL21(DE3) or **(B)** SHuffle^TM^ was transformed with pET15b (an empty vector) or pET15b harboring human SOD1(WT) cDNA, and the protein expression was induced with IPTG (see Materials and Methods). Results obtained by addition of 1 mM ZnSO_4_ at the induction of protein expression were also included in **(A)** (SOD1(WT) + 1 mM ZnSO_4_). Also, expression of SOD1 with C57/146S mutations, in which an intramolecular disulfide bond cannot form, was examined in *E. coli* SHuffle^TM^ and shown in **(B)**. Cell lysates were fractionated into soluble supernatant (s) and insoluble pellets (p), treated with iodoacetamide, and then analyzed with non-reducing SDS-PAGE by using a 15% polyacrylamide gel. White (SOD1^S-S^) and black (SOD1^SH^) arrows at the right side of the gel image indicate positions of bands corresponding to SOD1 with and without a disulfide bond, respectively.

Introduction of an intramolecular disulfide bond between Cys57 and Cys146 in SOD1 has been known to increase the electrophoretic mobility of SOD1 (Furukawa et al., 2004); therefore, thiol-disulfide status of SOD1 can be determined by non-reducing SDS-PAGE analysis. When overexpressed in *E. coli* BL21, wild-type human SOD1 (SOD1(WT)) was found to be in a disulfide-reduced form (SOD1^SH^) with a small fraction of a disulfide-form (SOD1^S-S^; **Figure 1A**). Also notably, most of SOD1^SH^ was insoluble, while SOD1^S-S^ remained soluble (**Figure 1A**). These results are consistent with a previous finding that SOD1^SH^ is highly prone to insoluble aggregation *in vitro* (Furukawa et al., 2008). Given that cytoplasm of *E. coli* provides a highly reducing environment (Hwang et al., 1992), formation of a disulfide bond in proteins will be an unfavorable process in the cytoplasm. Furthermore, metal-chelating capacity of bacterial cytoplasm has been reported to be extremely high (Outten and O’Halloran, 2001; Changela et al., 2003), where SOD1 is supposed to be in a metal-deficient apo state. Indeed, exogenous supplementation of ZnSO_4_ in a growth media increased the soluble fraction of overexpressed SOD1^SH/S-S^ (**Figure 1A**), supporting previous reports that binding of a zinc ion protects SOD1 from aggregation (Furukawa et al., 2008). In the reducing environment of cytoplasm, where metal-chelating capacity is also significant, SOD1 remain in a disulfide-reduced apo state and is prone to insoluble aggregation.

### OXIDIZING ENVIRONMENT PROTECTS SOD1 FROM AGGREGATION BY DISULFIDE FORMATION

To examine effects of disulfide formation on SOD1 aggregation *in vivo*, SOD1(WT) was overexpressed in *E. coli* SHuffle^TM^, where cytoplasmic reductive pathways are genetically diminished (Lobstein et al., 2012). More specifically, thioredoxin reductase (*trxB*) and glutathione reductase (*gor*) have been removed in *E. coli* SHuffle^TM^, which provides oxidizing cytoplasm and thus enables to introduce disulfide bonds in cytoplasmically expressed proteins. As shown in **Figure 1B**, SOD1(WT) overexpressed in *E. coli* SHuffle^TM^ was found to form a disulfide bond and remain soluble, showing that correct introduction of a disulfide bond in SOD1 can prevent its insoluble aggregation *in vivo*. When SOD1 with C57S/C146S mutations, in which a conserved disulfide bond (Cys57–Cys146) cannot form, was overexpressed in *E. coli* SHuffle^TM^, significant amounts of mutant SOD1 was again found in the insoluble fraction. This result hence emphasizes a protective role of the disulfide bond against aggregation of SOD1. In eukaryotes, a copper chaperone for SOD1 (CCS) has been shown to introduce the intramolecular disulfide bond in SOD1 (Furukawa et al., 2004), while CCS-independent pathway(s) for disulfide formation in SOD1 also appears to exist (Subramaniam et al., 2002; Leitch et al., 2009). Given no CCS homologues in bacteria, the results obtained by using SHuffle^TM^ (**Figure 1B**) implies that oxidizing environment is sufficient for introducing a correct disulfide bond into wild-type SOD1 even without CCS and thereby protecting the protein from being misfolded/aggregated.

### ALS MUTATIONS COMMONLY AGGRAVATE THE AGGREGATION PHENOTYPE OF SOD1 UNDER REDUCING ENVIRONMENT

**FIGURE 2:**
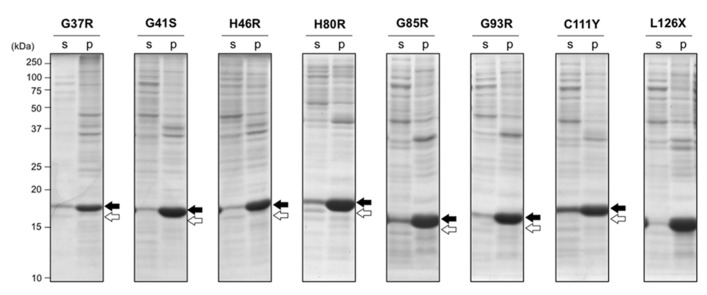
**Aggregation of disulfide-reduced SOD1 with fALS mutations in *E. coli* BL21 was not rescued by addition of ZnSO_4_**. *E. coli* BL21(DE3) was transformed with pET15b harboring human SOD1 cDNA with indicated fALS mutations, and the protein expression was induced by IPTG in the presence of 1 mM ZnSO_4_. Cell lysates were fractionated into soluble supernatant (s) and insoluble pellets (p), treated with iodoacetamide, and then analyzed with non-reducing SDS-PAGE by using a 15% polyacrylamide gel. White (SOD1^S-S^) and black (SOD1^SH^) arrows at the right side of the gel image indicate positions of bands corresponding to SOD1 with and without a disulfide bond, respectively.

To test effects of fALS-causing mutations on SOD1 aggregation *in vivo*, several types of mutant SOD1 were overexpressed in *E. coli* BL21. SOD1 with A4V mutation has been reported to be expressed in *E. coli* BL21 as insoluble pellets (Leinweber et al., 2004), and all of the other mutant SOD1s tested here were found as a disulfide-reduced form in insoluble pellets regardless of the absence (data not shown) or presence (**Figure 2**) of 1 mM ZnSO_4_ in a growth media. This is in sharp contrast to wild-type SOD1, which was expressed as a soluble form when cultured in a growth media supplemented with zinc ions (**Figure 1A**). It has been suggested that the decreased affinity of zinc ion is a common pathogenic denominator of fALS-mutant SOD1 (Goto et al., 2000; Hayward et al., 2002); therefore, supplementation of 1 mM ZnSO_4_ to a growth media is considered to be insufficient for metallation of mutant SOD1 in the *E. coli* cytoplasm. Also notably, significant amounts of SOD1^S-S^ were observed in a soluble fraction when the wild-type protein was expressed in *E. coli* BL21 (**Figure 1A**); however, in fALS-mutant SOD1s, formation of the disulfide bond was hardly observed (**Figure 2**). These results are consistent with previous reports showing increased susceptibility of the disulfide bond to reducing agents by pathogenic mutations (Tiwari and Hayward, 2003). Under the reducing environment with high metal-chelating capacity of *E. coli* cytoplasm, therefore, SOD1 is found to exhibit increased propensities for aggregation with fALS-causing mutations.

### FALS-MUTANT SOD1 TEND TO FORM DISULFIDE-LINKED OLIGOMERS UNDER OXIDIZING ENVIRONMENT

**FIGURE 3:**
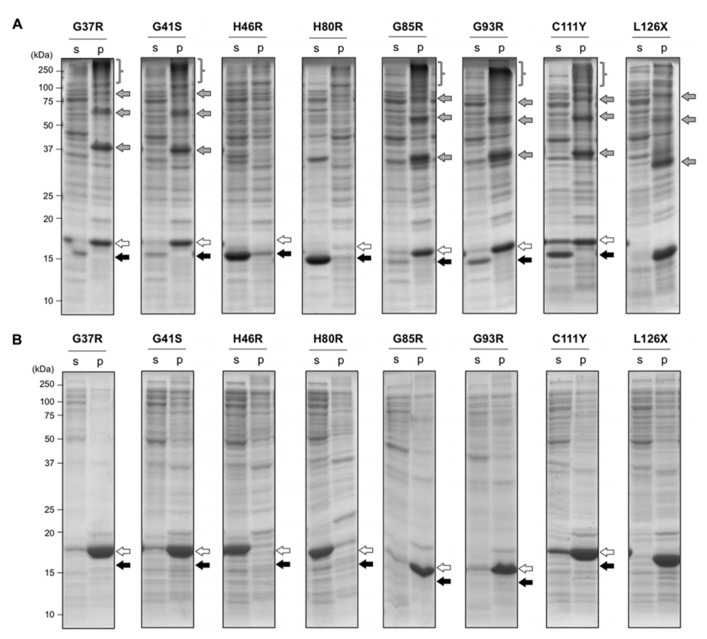
**Aggregation of mutant SOD1 in *E. coli* SHuffle^TM^ associates with the formation of inter-molecular disulfide crosslinks**. *E. coli* SHuffle^TM^ was transformed with pET15b harboring human SOD1 cDNA with indicated fALS mutations, and the protein expression was induced by IPTG. Cell lysates were fractionated into soluble supernatant (s) and insoluble pellets (p), treated with iodoacetamide, and then analyzed with **(A)** non-reducing or **(B)** reducing SDS-PAGE by using a 15% polyacrylamide gel. White (SOD1^S-S^) and black (SOD1^SH^) arrows at the right side of the gel image indicate positions of bands corresponding to SOD1 with and without a disulfide bond, respectively. In addition, gray arrows and braces in **(A)** represent the disulfide-linked SOD1 oligomers, which disappeared in reducing SDS-PAGE **(B)**.

In wild-type SOD1, the intramolecular disulfide bond was efficiently introduced under the oxidizing environment, which then protected the protein from aggregation (**Figure 1B**). In contrast, when several fALS-mutant SOD1s were expressed in the oxidizing cytoplasm of *E. coli* SHuffle^TM^, significant amounts of SOD1 were found to remain in insoluble pellets (**Figure 3**). More specifically, a monomer and higher-order oligomers of mutant SOD1 (G37R, G41S, G85R, G93R, C111Y, and L126X) were evident in an insoluble fraction, while SOD1s with H46R and H80R mutations were obtained as soluble proteins with an intramolecular disulfide bond (**Figure 3A**). When analyzed in reducing SDS-PAGE, furthermore, those higher-order oligomer bands were collapsed and merged to the monomer band (**Figure 3B**), indicating that the oligomers were formed *via* disulfide-crosslinks. Formation of intra- or inter-molecular disulfide bond is considered to depend upon intracellular concentration of SOD1 proteins, but **Figure 3B** shows similar expression levels of SOD1 proteins in *E. coli* examined here. In those mutant SOD1s forming disulfide-linked oligomers (G37R, G41S, G85R, G93R, C111Y, but not L126X), small amounts of proteins were also detected in a soluble fraction with an intramolecular disulfide bond. Notably, SOD1 with H46R and H80R mutations have been shown to exhibit thermostability comparable to that of the wild-type protein (Rodriguez et al., 2005; Vassall et al., 2011); therefore, fALS-causing mutations that significantly destabilize a native structure of SOD1 favor the formation of disulfide-crosslinked oligomers under oxidizing environment.

### MUTANT SOD1 FORMS AMYLOID-LIKE FIBRILLAR AGGREGATES IN *E. coli*

**FIGURE 4:**
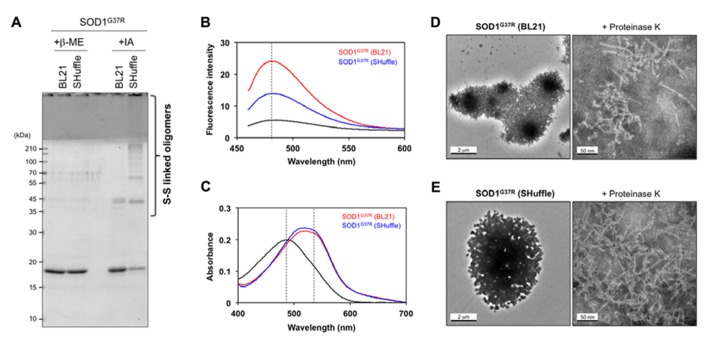
**Amyloid-like characters of SOD1 aggregates purified from insoluble inclusions in *E. coli***. **(A)** SOD1(G37R) aggregates purified from insoluble inclusions in *E. coli* were reacted with iodoacetamide for protection of free thiol groups and analyzed with reducing (+β-ME) and non-reducing (+IA) SDS-PAGE. Disulfide-crosslinked oligomers were identified in SOD1(G37R) aggregates purified from insoluble inclusions in *E. coli* SHuffle^TM^ but not in BL21(DE3). **(B, C)** Tinctorial properties of SOD1(G37R) aggregates purified from *E. coli* BL21 and SHuffle^TM^ (red and blue curves, respectively) were examined by **(B)** fluorescence of thioflavin T and **(C)** absorption of Congo red. Black curves represent **(B)** fluorescence spectrum of thioflavin T and **(C)** absorption spectrum of Congo red without addition of SOD1 aggregates. **(D, E)** Electron micrograms of SOD1(G37R) aggregates purified from *E. coli* **(D)** BL21 and **(E)** SHuffle^TM^. SOD1(G37R) aggregates exhibit large, amorphous morphologies (left panels), while fibrillar structures become evident after brief treatment of aggregates with Proteinase K (right panels). A bar represents 2 μm (left panels) or 50 nm (right panels).

To further characterize the SOD1 aggregates formed in *E. coli*, a protocol to purify those insoluble aggregates were first established, in which insoluble fractions of *E. coli* lysates were extensively washed with 1 M NaCl, 1% Sarkosyl, and then acetone (see Materials and Methods). When purified aggregates of SOD1(G37R) were reacted with iodoacetamide to protect free thiol groups and then analyzed in non-reducing SDS-PAGE, disulfide-linked oligomers were found to be successfully isolated from *E. coli* SHuffle^TM^ (**Figure 4A**, +IA). In contrast, purified aggregates of SOD1(G37R) from *E. coli* BL21 were mainly composed of disulfide-reduced monomers with slight contamination of dimers (**Figure 4A**, +IA). Furthermore, after extensive washes, both SOD1(G37R) aggregates from BL21 and SHuffle^TM^ produced a single band in reducing SDS-PAGE (+β-ME), supporting successful purification of the SOD1 aggregates.

Aggregates of SOD1 *in vitro* have been shown to have structural features similar to those of amyloid, which are characterized by fibrillar morphologies with β-sheet-rich structures (Furukawa et al., 2008). As shown in **Figure 4B**, both SOD1(G37R) aggregates purified from *E. coli* BL21 and SHuffle^TM^ were found to increase the thioflavin T fluorescence but with distinct intensity. In addition, those SOD1(G37R) aggregates were found to red-shift an electronic absorption spectrum of Congo red (**Figure 4C**). These tinctorial changes of thioflavin T and Congo red have been typically observed in protein aggregates with amyloid-like properties (Klunk et al., 1999; LeVine, 1999). It is, therefore, possible that SOD1(G37R) forms amyloid-like aggregates both in reducing (BL21) and oxidizing (SHuffle^TM^) environment, but its molecular structure might be dependent upon the redox environment where the aggregates form.

While amyloid-like aggregates generally exhibit fibrillar morphologies, SOD1(G37R) aggregates purified from insoluble inclusions in *E. coli* displayed not fibrils but amorphous, large lump-like structures under an electron microscope (**Figures 4D,E**, left panels). These structures are considered to be attained with aggregation of monomeric and disulfide-linked multimeric SOD1 proteins through SDS-sensitive interactions. When these inclusions were briefly treated with a non-specific protease, Proteinase K, however, a protease-resistant core of inclusions became exposed and was found to exhibit fibrillar morphologies with *approx*. 5.5 nm of the diameter (**Figures 4D,E**, right panels). While morphological differences of SOD1(G37R) aggregates were not clear between *E. coli* BL21 and SHuffle^TM^, exposure of fibrillar structures by treatment with proteases have been previously reported in *E. coli* inclusions of Aβ peptide (Morell et al., 2008) and HET-s (Sabate et al., 2009), which are known to be fibrillogenic. Taken together, therefore, this study successfully reproduces fibrillar aggregation of mutant SOD1 proteins in the cytoplasm of *E. coli* and further implies that intracellular redox environment could modulate the properties of SOD1 aggregates by changing its thiol-disulfide status.

### IMPLICATIONS TO PATHOLOGIES OF SOD1-RELATED fALS CASES

**FIGURE 5:**
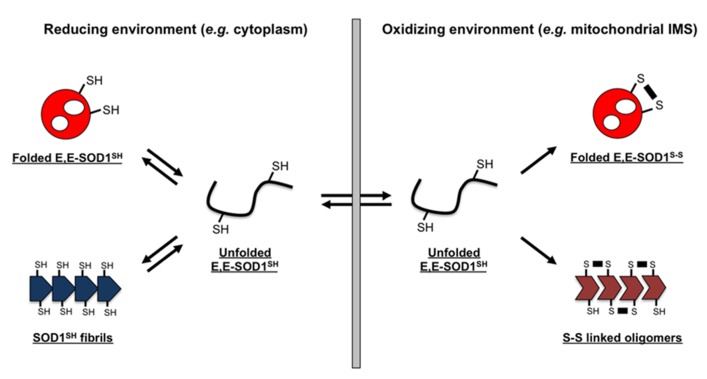
**Schematic representation of a redox-dependent SOD1 folding/misfolding model**. SOD1 exists as an apo and disulfide-reduced state (E,E-SOD1^SH^) and tends to form fibrillar aggregates (SOD1^SH^ fibrils) in reducing environment of the cytosol; in contrast, oxidizing environment stabilizes a soluble, folded state of SOD1 by introduction of an intramolecular disulfide bond (E,E-SOD1^S-S^). Many fALS mutations are considered to destabilize the structure of E,E-SOD1^SH^, increasing the fraction of misfolded state. In such a situation, oxidizing environment favors the formation of SOD1 oligomers cross-linked via abnormal disulfide bonds. Depending upon redox environment where SOD1 exists, several distinct forms in SOD1 aggregates are hence possible.

More than 70% of intracellular SOD1 has been detected in the cytoplasm (Chang et al., 1988), which is normally kept as reducing environment by maintaining high concentrations of reduced glutathione (Hwang et al., 1992). Indeed, mutant SOD1 proteins are abnormally accumulated in the cytoplasm as Lewy body-like hyaline inclusions (Shibata et al., 1996), and those insoluble forms of mutant SOD1 have been characterized as a disulfide-reduced state in SOD1-fALS model mice (**Figure 5**, left; Jonsson et al., 2006; Karch et al., 2009). These observations are consistent with the *in vitro* findings that inability to form the disulfide bond significantly increases the propensities of SOD1 for fibrillar aggregation (Furukawa et al., 2008). In reducing environment of the cytoplasm, the disulfide bond in SOD1 is introduced by a copper chaperone protein, CCS (Furukawa et al., 2004), and appears to be protected from the reductive cleavage by its burial at the dimer interface. Some fALS mutations would hence disturb the interaction with CCS and/or facilitates the monomerization of SOD1; thereby, stability of the disulfide bond becomes decreased, which then triggers the fibrillar aggregation of disulfide-reduced SOD1 in the cytoplasm (the disulfide-reduction model; Toichi et al., 2013).

In contrast to such disulfide-reduction model of SOD1 aggregation, little accumulation of cytoplasmic inclusions has been observed in some fALS model mice expressing mutant SOD1, albeit with significant mitochondrial pathologies (Nagai et al., 2001; Watanabe et al., 2001). *Approximately* 3% of total SOD1 is known to localize at the IMS of mitochondria (Okado-Matsumoto and Fridovich, 2001), where the redox potential is significantly more oxidizing than that of the cytoplasm (Hu et al., 2008). Furthermore, mitochondria of a motor neuron, which is the most damaged cell in fALS cases (Bruijn et al., 2004), have been reported to provide more oxidizing environment than those of the other types of cells (Ferri et al., 2006). Given that reduction of a disulfide bond in SOD1 is required for its transport into mitochondrial IMS (Field et al., 2003), disulfide-reduced SOD1 just after transported is considered to experience significantly oxidizing environment of motorneuronal IMS (**Figure 5**, right). Transported SOD1 polypeptide then folds into the native three-dimensional conformation, and the intramolecular disulfide bond is introduced by mitochondrial CCS. The folding process of SOD1 is, however, deterred by fALS-causing mutations (Nordlund and Oliveberg, 2006; Bruns and Kopito, 2007), which would increase the chance to form aberrant disulfide crosslinks under oxidizing environment of the mitochondrial IMS (**Figure 5**, right). Indeed, SOD1 oligomers crosslinked *via* disulfide bonds have been observed in mitochondria isolated from spinal cords of fALS-model mice expressing mutant SOD1 (Deng et al., 2006). Also in cultured motorneuronal cells, disulfide-crosslinked oligomers of mutant SOD1 have been reproduced in mitochondria but not in cytoplasm (Ferri et al., 2006). In the oxidizing environment of organelles, therefore, disulfide-reduced mutant SOD1 is highly prone to crosslinking *via* abnormal disulfide bonds, which will not occur under reducing environment of the cytosol.

So far, it has been well known that misfolding/aggregation of mutant SOD1 occurs both in cytoplasm and mitochondrial IMS; however, any possible differences in the molecular properties of SOD1 aggregates between those two cellular compartments have been hardly noticed. An intracellular folding process of protein molecules into their native conformations has been well known to be significantly affected or even controlled by chaperone proteins, but motorneurons are equipped with different sets of chaperones from those in *E. coli*; therefore, a pathological process of SOD1 aggregation in SOD1-related fALS patients, which in general gradually proceed over several decades, would not be precisely reproduced in the *E. coli* overexpression system examined here. Nonetheless, distinct forms of misfolded/aggregated SOD1, especially in terms of their thiol-disulfide status, appear to be possible that depend upon the redox environment surrounding SOD1 proteins (**Figure 5**). While it is further required to test if distinct properties of those SOD1 aggregates include toxicities to motorneurons, the redox-dependent aggregation of mutant SOD1 proteins would be one of the molecular mechanisms describing pathological heterogeneity observed in SOD1-related fALS cases.

## MATERIALS AND METHODS

### PLASMIDS, *E. coli* STRAINS AND PROTEIN EXPRESSION

A vector, pET15b (Novagen), was used for the construction of plasmids expressing human SOD1 without any tags; a SalI site was first introduced between BamHI and Bpu1102I sites of pET15b, and cDNA of human SOD1 was then cloned between NcoI and SalI site. Mutations were introduced by an inverse PCR method using KOD-FX-neo DNA polymerase (TOYOBO), and all constructs used in this study were confirmed by DNA sequencing. Competent cells** of *E. coli*, BL21(DE3; New England Biolabs) and SHuffle^TM^ T7 Express lysY (New England Biolabs), were transformed with a plasmid. *E. coli* cells harboring a plasmid were cultured in Luria-Broth media containing 50 mg/L ampicillin (LB/Amp) by shaking at 200 rpm, and the expression of SOD1 proteins was induced with 1 mM isopropyl 1-thio-β-D-galactopyranoside at 37°C for 6 h. To test effects of Zn^2^^+^ ions on SOD1 aggregation, 1 mM ZnSO_4_ was further added at the induction of protein expression.

### ELECTROPHORETIC ANALYSIS OF SOD1 PROTEINS EXPRESSED IN *E. coli*

*E. coli* cells cultured in 5 mL LB/Amp media were collected by centrifugation (2,000 × *g*, 10 min) and lysed by ultrasonication in 100 μL of PBS with 2% Triton X-100 and 100 mM iodoacetamide. Soluble supernatant and insoluble pellets were obtained with centrifugation of cell lysates (20,000 × *g*, 10 min), and insoluble pellets were further solubilized by ultrasonication in 100 μL of PBS with 2% SDS and 100 mM iodoacetamide. The soluble supernatant and the re-solubilized insoluble pellets were incubated at 37°C for 30 min, which ensures the protection of thiol groups by modification with iodoacetamide. 0.8 (BL21) or 1.5 (SHuffle^TM^) μL of the samples were then mixed with an SDS-PAGE sample buffer, and 10% β-mercaptoethanol was further added for reducing SDS-PAGE. The samples were boiled at 100°C for 5 min, electrophoresed on a 15% SDS-PAGE gel, and then stained with Coomassie brilliant blue.

### PURIFICATION OF SOD1 AGGREGATES FROM *E. coli*

Insoluble pellets obtained by cell lysis in PBS/2% Triton X-100 were re-suspended in 1 M NaCl/H_2_O with ultrasonication and centrifuged at 20,000 × *g* for 10 min to collect insoluble pellets. After washed again by 1 M NaCl, the insoluble pellets were washed three times with PBS/1% Sarkosyl and then washed with cold acetone. After dried up with SpeedVac (Savant), the pellets were re-suspended in 100 mM Na-Pi/100 mM NaCl/5 mM EDTA, pH 7.4 with ultrasonication. Purified pellets were analyzed by SDS-PAGE after being re-dissolved in PBS/2% SDS/100 mM iodoacetamide and loaded on a 15% polyacrylamide gel in the presence and absence of 10% β-mercaptoethanol (**Figure 4A**). Monomer-based concentration of SOD1 aggregates purified from inclusions was spectroscopically determined from the absorbance at 280 nm in 6 M guanidine hydrochloride using 5,500 cm^-^^1^M^-^^1^ as an extinction coefficient.

### CHARACTERIZATION OF SOD1 AGGREGATES FROM *E. coli*

To test if SOD1 aggregates exhibit amyloid-like tinctorial properties, thioflavin T assay was performed. 10 μM (monomer-based) SOD1 aggregates purified from *E. coli* inclusions were mixed with 25 μM thioflavin T in 100 mM Na-Pi/100 mM NaCl/5 mM EDTA, pH 7.4, and fluorescence spectra excited at 442 nm were measured from 460 to 600 nm by using F-4500 (Hitachi). Congo red assay was also performed to test amyloid-like properties in SOD1 aggregates. 3 μM (monomer-based) SOD1 aggregates purified from *E. coli* inclusions were mixed with 5 μM Congo red in 100 mM Na-Pi/100 mM NaCl/5 mM EDTA, pH 7.4, and absorption spectra were measured by UV-2400PC (Shimadzu).

Morphologies of SOD1 aggregates formed in *E. coli* were examined by electron microscopy. Insoluble inclusions purified from *E. coli* were adsorbed on STEM100Cu grids coated by elastic carbon (Okenshoji), washed with water, and then negatively stained with 2% phosphotungstic acid. Images were obtained using an electron microscope (Tecnai^TM^ Spirit, FEI). 500 μL of *approximately* 100 μM (monomer-based) SOD1 aggregates purified from insoluble *E. coli* inclusions were mixed with 2 μL of 5 mg/mL Proteinase K and incubated at 37°C for 10 min. After ultracentrifuged at 110,000 × *g* for 15 min, insoluble pellets were washed with 500 μL of water and again ultracentrifuged. Resultant pellets were re-suspended in 100 μL of water and observed by an electron microscope as mentioned above.

## Conflict of Interest Statement

The author declares that the research was conducted in the absence of any commercial or financial relationships that could be construed as a potential conflict of interest.
